# Investigation of Ring and Star Polymers in Confined Geometries: Theory and Simulations

**DOI:** 10.3390/e23020242

**Published:** 2021-02-19

**Authors:** Joanna Halun, Pawel Karbowniczek, Piotr Kuterba, Zoriana Danel

**Affiliations:** 1 Institute of Nuclear Physics, Polish Academy of Sciences, 31-342 Cracow, Poland; 2 Institute of Physics, Cracow University of Technology, 30-084 Cracow, Poland; pkarbowniczek@pk.edu.pl (P.K.); zoriana.danel@pk.edu.pl (Z.D.); 3 Faculty of Physics, Astronomy and Applied Computer Sciences, Jagiellonian University in Cracow, 30-348 Cracow, Poland; piotr.kuterba@uj.edu.pl

**Keywords:** critical phenomena, surface effects, renormalization group, polymers

## Abstract

The calculations of the dimensionless layer monomer density profiles for a dilute solution of phantom ideal ring polymer chains and star polymers with f=4 arms in a Θ -solvent confined in a slit geometry of two parallel walls with repulsive surfaces and for the mixed case of one repulsive and the other inert surface were performed. Furthermore, taking into account the Derjaguin approximation, the dimensionless layer monomer density profiles for phantom ideal ring polymer chains and star polymers immersed in a solution of big colloidal particles with different adsorbing or repelling properties with respect to polymers were calculated. The density-force relation for the above-mentioned cases was analyzed, and the universal amplitude ratio B was obtained. Taking into account the small sphere expansion allowed obtaining the monomer density profiles for a dilute solution of phantom ideal ring polymers immersed in a solution of small spherical particles, or nano-particles of finite size, which are much smaller than the polymer size and the other characteristic mesoscopic length of the system. We performed molecular dynamics simulations of a dilute solution of linear, ring, and star-shaped polymers with N=300 , 300 (360), and 1201 (4 × 300 + 1-star polymer with four arms) beads accordingly. The obtained analytical and numerical results for phantom ring and star polymers are compared with the results for linear polymer chains in confined geometries.

## 1. Introduction

The investigation of polymer adsorption on surfaces, as well as polymer-colloid mixtures or polymer solutions with nano-particles has attracted much interest during the last few decades because of the broad practical application in materials science technology and medicine in the context of better understanding of drug delivery systems, as well as for biological and fundamental reasons such as the prevention of virus fusion with host cells [1,2,3,4,5]. We can distinguish two cases that usually lead to qualitatively different results during the analysis of the microscopic interactions in polymer-colloid or in polymer nano-particle mixtures. The first case results from the situation when polymers can adsorb on the colloidal or nano-particles, and it leads to the protection of particles from flocculation and to the stabilization of suspensions [6,7]. In the second case, we have to deal with a mixture of colloidal or nano-particles and free nonadsorbing polymers, which results in the so-called depletion effect [8] when polymers are expelled from the region between two particles or between a particle and a surface of the wall due to entropic reasons. In this case, an unbalanced pressure from the outside pushes colloidal particles or nano-particles to a wall or two particles towards each other. In such a situation, the key role is played by the depletion potential, which gives rise to the depletion force between colloidal particles or nano-particles, as well as between particles and a surface of the wall, respectively. We can assume that the magnitude of this depletion force depends on the concentration of polymer solutions, the effective size of polymer coils, the topology of polymer chains, as well as the size and the shape of the colloidal or nano-particles and the width of their separation. In a series of papers [8,9,10], the measurements of the depletion force, which arises between a wall and a single particle immersed in a dilute solution of nonionic polymer chains in a good solvent, were performed. The depletion force enters into a complex force balance with other intercolloidal interactions, such as Derjaguin, Landau, Vervey and Overbeek theory (DLVO), hydrophobic interaction, and hydration [4,5,11]. During a long period, the interaction between linear polymers and colloidal particles was investigated in the framework of an idealized physical model of non-deformable hard spheres proposed by Asakura and Oosawa [12,13]. The problem is that the common Asakura–Oosawa approximation modeling the polymer coils as hard spheres appears to be virtually inadequate for small particles and fails by about 10% for large particles because long polymers are flexible objects and cannot be modeled as hard spheres. One should also bear in mind that flexible linear polymer chains interacting with colloidal particles have been investigated in the framework of the phenomenological scaling theory [14,15,16,17], by means of integral equation techniques [18,19,20], via the self-consistent field theory [21,22], a dimensionally regularized continuum version of the field theory with minimal subtraction of poles in ϵ -expansion, where ϵ=4−d (*d* is the dimensionality of space) [23,24,25,26], and in a series of our papers [27,28,29,30,31] via the massive field theory approach and Derjaguin approximation [32] for the case of two walls with repulsive surfaces, two walls with inert surfaces, and for the mixed situation of one repulsive and the other inert surface.

Our results obtained in [27] for a dilute solution of linear polymer chains showed that the depletion force is attractive in both cases: for the slit of two parallel walls with repulsive surfaces and for one repulsive and the other inert surface (where the adsorption threshold occurs). The value of the depletion force in the case of one repulsive and the other inert surface is smaller than in the case of two repulsive surfaces, and in the case of two inert surfaces, the depletion force becomes repulsive in the case of a dilute solution of real linear polymer chains with the excluded volume interaction (EVI) in a good solvent. This result is very important for practical reasons, because it means that in such systems, we observe the reduction of the static friction, and as a result, such systems can be used for producing new types of nano- and micro-mechanical devices. The calculation of the depletion force was performed by numerical methods [33,34] using the model of random walk (RW) for ideal linear polymer chain in a Θ -solvent and the model of self-avoiding walks (SAWs) for a real polymer chain with the EVI in a good solvent confined inside the slit of two repulsive walls. The validity of the universal density-force relation proposed by Joanny, Leibler, and de Gennes [14] for the different cases of a dilute solution of linear polymer chains in confined geometries, as well as for the case of a semi-dilute solution of free linear polymers in a half space and for the case of polymer in a half space containing a mesoscopic colloidal particle of arbitrary shape was confirmed in [24]. The above-mentioned universal density-force relation was verified by simulation methods using an off-lattice bead-spring model of a polymer chain trapped between two parallel repulsive walls [33] and by the lattice Monte Carlo (MC) algorithm on a regular cubic lattice in three dimensions [34]. It should be mentioned that in a series of our papers [28,29], the universal density-force relation for a dilute solution of linear ideal and real polymers with the EVI in a good solvent immersed in a slit geometry of two parallel walls with repulsive surfaces, one repulsive and the other inert surface, as well as in the case of a dilute polymer solution of linear polymer chains confined in a semi-infinite space containing the mesoscopic spherical colloidal particle of big radius was analyzed by analogy, as was proposed by Eisenriegler [24], and the corresponding universal amplitude ratio was obtained in the framework of the massive field theory approach directly in fixed space dimensions d=3 up to one-loop order. The interaction of long flexible nonadsorbing linear polymers with mesoscopic colloidal particles of big and small size and a different shape was the subject of a series of papers [25,35]. The obtained results for long flexible linear polymer chains indicate that focusing on such systems leads to universal results that are independent of microscopic details and are free of non-universal model parameters and depend only on the shapes of particles and the ratios of three characteristic lengths of the system such as the radius of the particle, the polymer size, and the distance between the particle and the wall or between two particles, respectively.

In a series of the atomic force spectroscopy (AFM) experiments [36,37], it was shown that the biopolymers such as DNA very often present a ring topology. One of the examples is that of *Escherichia coli* (*E. coli*) bacteria, which is not a linear polymer, but has a ring topology [38], and on the other hand, it is resistant to a wide spectrum of antibiotics. The biopolymers of the DNA of some viruses such as bacteriophages λ that infect and destroy *E. coli* bacteria oscillate between a linear and ring topology, as was shown in a series of papers [39,40]. The linear form of DNA is encountered in mature viruses; however, the inside of the host cell DNA of bacteriophages adopts a ring topology [41]. Such an investigation is important in the context of understanding the processes that lead to the destruction of *E. coli* bacteria with the help of bacteriophages λ and in the context of creating phage-therapy, which is an alternative way to antibiotic treatment, especially in the case of bacteria that demonstrate high resistance to antibiotics. Confinement and chain topology play a significant role in the shaping of individual chromosomes and in the process of their segregation, as was shown in [42].

It should be mentioned that the investigation of the statistical mechanical properties of ring polymer chains was the subject of a series of papers (see [31,37,40,43,44,45,46,47,48,49,50] and the literature cited there). The mean-squared radius of gyration and the scaling behavior in the average size for ring polymers for several nontrivial knots such as $0_{1},3_{1},4_{1},3_{1}\#3_{1}$ , and $3_{1}\#4_{1}$ were evaluated in [51]. The ring polymers with more complex knots with a finite number of monomers *N* are more compact and have a smaller radius of gyration, and this decreases their ability to spread out under confinement, as was established in [44]. In accordance with this, such investigations are important for the polymers with a long, but finite number of monomers, especially in the case of oligomers. On the other hand, in the limit N→∞ , the difference in the average size of ring polymers with different knots decreases relatively with the increasing of the degree of polymerization proportionally to $N^{1/\nu }$ , where ν is the Flory exponent. The numerical results based on the MC simulations [47] suggest that the knotted ring polymers will exert higher entropic forces on the walls of the confining slit than unknotted or linear polymers. The molecular dynamics simulations performed in [40] allowed obtaining the entropic force exerted on the walls arising from the confinement to a slit of a knotted ring and the monomer density profiles in the case of two repulsive walls. The results obtained in [40] showed that in the case of a narrow slit, more complex knot types in a ring polymer exert higher forces on the confining walls of the slit in comparison to unknotted polymer chains of the same length, and for relatively wide slits, the opposite situation takes place. Recently, advanced MC simulation techniques [52] were used in order to study the effect of nanoslit confinement on the topological properties of the circular model DNA, which was modeled as a semiflexible polymer chain. They showed that the knotting probability has strong slit width dependence. The investigation of the influence of topological constraints on the free energy and metric properties of an ideal ring polymer without excluded volume effects or attractive interactions confined in a narrow slit were performed in [53] using off-lattice MC simulations. The occurrence and behavior of polymer knots were studied extensively in a series of recent papers based on MC simulations [54,55,56,57]. For example, in [54], numerical simulations were used in order to show that the effective stiffening of DNA by the nematic arrangement promotes the formation of torus knots in phage capsids. It should be also noted that the conservation of the knot topology in simulations of ring polymers depends on the knotting probability of the knot, as was shown in [57] by the use of the self-avoiding polygons (SAPs) method.

Computational investigations of the good solvent solution properties of knotted rings with minimal crossing number $m_{c}$ in the range between zero and nine, as well as in the case of star polymers with the number of arms in the region between two and 20 were performed by combining MD simulation and path-integral calculations [58]. The basic configuration properties, i.e., the radius of gyration, hydrodynamic radius, as well as intrinsic viscosity, were analyzed [58], and the performed simulations indicated that the configurational properties of knotted rings and star polymers in a good solvent show a similar decrease with the increasing minimal crossing number and the number of arms of the star polymers. Therefore, in accordance with this, the comparison of the analytical calculation results of the statistical properties of ring polymers and star polymers is a very interesting task. It should be mentioned that deeper understanding of the statistical and conformation properties of star polymers is important since it is connected with the investigation of micellar and other polymeric surfactant systems [59,60], as well as networks [61,62]. As discussed in one of the latest reviews [63], the star polymers have a wide application for the production of new functional materials and can be used in nanotechnology and biological sciences as drug, gene, or siRNA/DNA vectors [63].

The series of our recent papers [31,64,65] connected with the investigation of the influence of the polymer chain topology on the depletion interaction potentials and the depletion forces indicate that polymer chains with the ring topology behave completely in a different way than linear polymer chains in confined geometries. For example, we obtained in [65] that in the case of a dilute solution of ideal ring polymer chains in a slit geometry of two parallel walls with mixed boundary conditions, the depletion force is repulsive in contrast to the case of a dilute solution of linear polymer chains (see [27]). The explanation of the above-mentioned result for the depletion force can be connected with the assumption that topological effects in this case start to play a crucial role. In this context, it is very important to investigate the monomer density profiles for a dilute solution of ring polymer chains in a slit geometry of two parallel walls with both repulsive surfaces and especially in the case of mixed boundary conditions with one repulsive and the other inert surface where the adsorption threshold occurs. On the other hand, it is interesting to investigate the behavior of a dilute solution of ring polymer chains and star polymers immersed in a solution of mesoscopic colloidal particles of a big size or nano-particles with a small, but finite size with different adsorbing or repelling properties with respect to polymers and discuss the validity of the density force relation in this case. As mentioned above, the other interesting task is the comparison of the analytical calculation of the statistical properties of ring polymers with different topologies and star polymers with different numbers of arms and with the same molecular mass. Unfortunately, the analytical understanding of the processes that take place in the case of immersing a dilute solution of ring polymer chains and star polymers in confined geometries with mixed boundary conditions is still incomplete and requires deeper investigation, especially in the context of the monomer density profiles and the depletion force calculations. In accordance with this, the above-mentioned investigation is the subject of the present paper.

## 2. The Model and the Polymer-Magnet Analogy

We consider a dilute solution of ring polymer chains confined in a slit geometry of two parallel walls with repulsive surfaces or mixed walls when one wall has a repulsive surface and the other one has an inert one and allow for the exchange of polymer coils between the slit and the reservoir. In such a situation, the polymer solution in the slit is in equilibrium contact with an equivalent solution in the reservoir outside the slit. We assume that the solution of polymers is sufficiently dilute; thus, the interchain interactions and the overlapping between different polymers can be neglected, and it is sufficient to consider the behavior of a single polymer chain. As is known, the behavior of a long flexible ideal polymer chain can be described by the model of a random walk (RW) on a regular lattice, and it corresponds to the case of the Gaussian ideal chain in a Θ -solvent [15,16,17,66,67]. The behavior of a real polymer chain with the excluded volume interaction (EVI) for the temperatures above the Θ -point can be described by the model of self-avoiding walks (SAWs) [15,16,17]. The situation when the solvent temperature is below the Θ -temperature corresponds to a poor solvent condition where polymer coils tend to collapse [68,69].

As was noted some time ago by de Gennes [15,16,17] and by Barber et al. [70], there is a formal analogy of the polymer adsorption problem to the equivalent problem in the semi-infinite $\phi^{4}$ O(n) -vector model of a magnet with a free surface [71,72,73]. Taking into consideration the well-known correspondence between the statistics of long flexible polymer chains and the critical behavior of magnetic systems developed by de Gennes [15,16,17] we can use powerful field theory methods and techniques for the investigation of polymer problems. Taking into account the Derjaguin approximation [32] and small sphere expansion [74,75,76], we consider the interaction of ring polymers with big mesoscopic colloidal particles and small colloidal or nano- particles of fixed size and show how the methods of field theory with boundaries [27,77] allow explaining the basic properties of polymer-colloid mixtures and polymer-induced interactions between the particles. As was mentioned by Eisenriegler [76], even in the case of ideal chains, integrating out the polymer degrees of freedom is a nontrivial task in the presence of the colloidal particles, and in accordance with this, the application of the field theory methods and techniques is very useful.

One of the most convenient models for analytical calculations is an Edwards-type model for a dilute polymer solution, which allows for an expansion in terms of the excluded volume interactions and conformable for a field-theoretical treatment via the polymer-magnet analogy developed by de Gennes [15,16,17]. The configuration of one polymer in the framework of this model is given by a path x(s) in *d*-dimensional space $V\in {ℜ}^{d}$ parametrized by a surface variable $0\leq s\leq L_{0}$ in the continuum limit when the length of each step is decreasing l→0 with a fixed “Gaussian surface” $L_{0}=Nl^{2}$ , where $L_{0}$ has the dimension of length squared and is proportional to the degree of polymerization (total number of monomers) *N* of the polymer chain. The Hamiltonian *H* of the model is given by:

(1) $$\frac{H(x(s))}{k_{B}T}=\frac{1}{2}\int_{0}^{L_{0}}ds{\left(\dot{x}\left(s\right)\right)}^{2}+\int_{V}d^{d}xU\left(x\right)\rho \left(x\right)+H_{int}\left(x\left(s\right)\right),$$

where $\dot{x}\left(s\right)=\frac{dx(s)}{ds}$ and $H_{int}$ allows describing the interactions between any two monomers of a real polymer chain with the EVI potential $U_{2}\left(x,x^{\prime }\right)$ . Our analytical calculations are concentrated on the investigation of the Gaussian ideal ring and star polymers in a Θ -solvent confined in a slit geometry of two parallel walls with different boundary conditions. The second term in (1) corresponds to one-body interaction potential U(x) , where $U\left(x\right)\to U\left(z\right)-\bar{\mu }\left(x\right)$ and $\bar{\mu }\left(x\right)$ is a chemical potential. As was noted by de Gennes [15,16,17], in the continuum limit l→0 , we can assume: U(z)=cδ(z) , for z≥0 , where the value *c* corresponds to the adsorption energy divided by $k_{B}T$ (or the surface enhancement constant in the field theoretical treatment). It should be mentioned that $\rho \left(x\right)=\int_{0}^{L_{0}}ds\delta \left(x-x(s)\right)$ is the monomer density at point x . In the framework of this formalism, the partition function is calculated as a functional integral (see Appendix A):

(2) $$Z_{c}\left(x^{\prime },L_{0}|x,0\right)=\int D\left[x\left(s\right)\right]e^{-\frac{H(x(s))}{k_{B}T}},$$

where $x\left(L_{0}\right)=x^{\prime }$ , x(0)=x , and x(s)∈V . The symbol D[x(s)] includes normalization such that $Z_{c}\left(x^{\prime },L_{0}|x,0\right)=1$ for the Gaussian ideal chain without interaction $H_{int}$ . The partition function (2) satisfies the boundary conditions:

(3) $$\partial_{z}Z_{c}\left(x^{\prime },L_{0}|x,0\right){{|}_{z=0}=cZ_{c}\left(x^{\prime },L_{0}|x,0\right)|}_{z=0}.$$

Taking into account the polymer-magnet analogy developed by de Gennes [15,16,17], the continuous chain model (1) can be mapped onto a corresponding field theory by a Laplace transform in the Gaussian variables $L_{0}$ to conjugate chemical potentials $\mu_{0}$ :

(4) $$G^{\left(2\right)}\left(x,x^{\prime }\right)=\int_{0}^{\infty }dL_{0}e^{-\mu_{0}L_{0}}Z_{c}\left(x^{\prime },L_{0}|x,0\right).$$

On the other hand, the Laplace transformed function $G^{\left(2\right)}\left(x,x^{\prime }\right)$ can be expressed as the n→0 limit of the functional integral over vector fields $\vec{\phi }\left(x\right)$ with *n* components $\phi_{i}\left(x\right)$ , i=1,…,n and x=(r,z) :

(5) $$G^{\left(2\right)}\left(x,x^{\prime }\right)=\int D\left[\vec{\phi }\left(x\right)\right]e^{-H[\vec{\phi }]},$$

with the Landau–Ginzburg–Wilson Hamiltonian $H(\vec{\phi })$ describing the system in the semi-infinite ( j=1 ) or confined geometry of two parallel walls ( j=1,2 ):

(6) $$\begin{array}{c} H\left[\vec{\phi }\right]=\int d^{d}x\left\{\frac{1}{2}{\left(\nabla \vec{\phi }\right)}^{2}+\frac{{\mu_{0}}^{2}}{2}{\vec{\phi }}^{2}\right\}\\ +\sum_{j=1}^{2}\frac{c_{j_{0}}}{2}\int d^{d-1}r{\vec{\phi }}^{2}, \end{array}$$

where the conjugate chemical potential $\mu_{0}$ is the “bare mass” in field-theoretical treatment. The deviation from the adsorption threshold is characterized by the value $c\propto \left(T-T_{a}\right)/T_{a}$ (where $T_{a}$ is the adsorption temperature), which changes sign at the transition between the adsorbed (the normal transition, c<0 ) and the non-adsorbed state (ordinary transition, c>0 ) [28,77]. The value *c* plays the role of a critical parameter. The value 1/N plays the role of a critical parameter analogous to the reduced critical temperature in magnetic systems. The multicritical phenomenon takes place at the adsorption threshold for long flexible infinite polymer chains, where 1/N→0 and c→0 .

The two-point correlation function $G_{\mu_{0},c}\left(x,x^{\prime }\right)$ can be interpreted as the usual correlation function $<\vec{\phi }\left(x\right)\vec{\phi }\left(x^{\prime }\right)>$ of the model Equation (6). We assume that the walls in a slit are located at the distance *L* from one another in the *z*-direction such that the surface of the bottom wall is located at z=0 and the surface of the upper wall is located at z=L. The surfaces of the system are characterized by a certain surface enhancement constant $c_{j_{0}}$ , where j=1,2 .

The interaction between ring polymer chain and the surfaces of the walls is implemented by the different boundary conditions. In the case of two walls with repulsive surfaces (where the segment partition function and thus the partition function for the whole polymer chain tends to zero as any segment approaches the surface of the walls), Dirichlet-Dirichlet boundary conditions (D-D b.c.) take place:

(7) $$\vec{\phi }\left(r,0\right)=\vec{\phi }\left(r,L\right)=0\;or\;c_{1}\to +\infty ,\;c_{2}\to +\infty ,$$

and for the mixed case of one repulsive and one inert surface, the Dirichlet–Neumann boundary conditions (D-N b.c.) are:

(8) $$\vec{\phi }\left(r,0\right)=0,\;\frac{\partial \vec{\phi }\left(r,z\right)}{\partial z}{|}_{z=L}=0\;or\;c_{1}\to +\infty ,\;c_{2}=0.$$

Thus, the partition function $Z(x,x^{\prime })$ of a single polymer chain with two ends fixed at x and $x^{\prime }$ is connected with the two-point correlation function $G^{\left(2\right)}\left(x,x^{\prime }\right)=<\vec{\phi }\left(x\right)\vec{\phi }\left(x^{\prime }\right)>$ of the order parameter densities ϕ(x) and $\phi (x^{\prime })$ in a Ginzburg–Landau–Wilson model via the inverse Laplace transform $\mu_{0}^{2}\to L_{0}$ :

(9) $$Z\left(x,x^{\prime };N,v_{0}\right)={\mathrm{IL}}_{\mu_{0}^{2}\to L_{0}}\left(<\vec{\phi }\left(x\right)\vec{\phi }\left(x^{\prime }\right)>{|}_{n\to 0}\right)$$

in the limit, where the number of components *n* tends to zero.

The fundamental two-point correlation function of the free theory corresponding to the effective Landau-Ginzburg-Wilson (LGW) Hamiltonian (6) in a mixed momentum-space (p,z) representation is:

(10) $$G_{ij}^{\left(2\right)}\left(p,p^{\prime };z,z^{\prime }\right)={\left(2\pi \right)}^{d-1}\delta_{ij}\delta \left(p+p^{\prime }\right)\;{\tilde{G}}_{\|}\left(p;z,z^{\prime };\mu_{0},c_{1_{0}},c_{2_{0}},L\right)\;,$$

where the free propagator ${\tilde{G}}_{\|}\left(p;z,z^{\prime };\mu_{0},c_{1_{0}},c_{2_{0}},L\right)$ of the model Equation (6) was obtained in [27] and is presented in Appendix B.

In the present paper, we focus our attention on the investigation of a dilute solution of phantom ring polymer chains, i.e., ring polymer chains where we perform the summation over all possible knot structures. In the case of a ring polymer chain, the ends x=(r,z) and $x^{\prime }=\left(r^{\prime },z^{\prime }\right)$ in the partition function Equation (9) should coincide. In accordance with this, we assume that $x^{\prime }=x+\Delta x$ and consider the limit Δx→0 . It should be mentioned that Δx is proportional to *l*, and taking into account that we consider a continuous limit, when the length of each step is decreasing l→0 , our assumption Δx→0 is realistic. As was shown by Eisenriegler [76,78], the scaling behavior of the phantom ring polymer chain partition function with $x=x^{\prime }=0$ in the infinite space in the limit N→∞ is characterized by the negative power law exponent:

$$Z\left(0,0;N\right)∼e^{Nl^{2}t_{c}}N^{-d\nu },$$

because *d* and ν have positive values and demonstrate different behavior than the linear polymer chain.

The most common parameter in polymer physics to denote the polymer chain size that is observable in experiments is the radius of gyration $R_{g}$ . For example, for linear polymer chains, the following relation takes place: $<R_{g}^{2}>\;=\chi_{d}^{2}\frac{<R_{x}^{2}>}{2}$ , where $\chi_{d}$ is a universal numerical prefactor depending on the dimension *d* of the system (see Refs. [78,79,80]), and $R_{x}$ is the projection of the end to end distance R onto the direction of the *x* axis. In the case of ideal polymer chains, one has $\chi_{d}^{2}=\frac{d}{3}$ , and for d=3 , in the case of ideal ring polymer chains, the following relation takes place: $<R_{g}^{2}>=\frac{Nl^{2}}{12}$ , which can be rewritten formally in the form $<R_{g}^{2}>\to <R_{x}^{2}>/4$ in order to keep similar notations as for linear polymer chain [65]. The mean-squared radius of gyration $R_{g}$ for star polymer chains is [81]:

(11) $$<R_{g}^{2}>=\frac{Nl^{2}}{6f}\left(3-\frac{2}{f}\right),$$

and can be rewritten respectively in the form: $<R_{g}^{2}>\to \frac{5}{16}<R_{x}^{2}>$ .

## 3. The Monomer Density and the Density-Force Relation for Ring Polymers

We consider the layer monomer densities $\rho_{\lambda }\left(\tilde{z}\right)$ defined by:

(12) $$\rho_{\lambda }\left(\tilde{z}\right)d\tilde{z}=\frac{{\left(2R_{g}\right)}^{1/\nu }}{N}dN_{\lambda }\left(\tilde{z}\right),$$

where the value $dN_{\lambda }\left(\tilde{z}\right)$ means the number of monomers in the layer between $\tilde{z}$ and $\tilde{z}+d\tilde{z}$ . Furthermore, $\rho_{\lambda }\left(\tilde{z}\right)$ is obtained from monomer density $\rho (\tilde{r},\tilde{z})$ after integration over the d−1 components parallel to the wall [24]. The scaling dimensions of $\rho (\tilde{r},\tilde{z})$ are $l^{1/\nu -d}$ and equal the ordinary dimensions of the quantity:

(13) $$\Psi \left(\tilde{x}\right)=\frac{{\left(2R_{g}\right)}^{1/\nu }}{2L_{0}}\Phi^{2}\left(\tilde{x}\right).$$

In general, the monomer density of a single polymer chain trapped between two parallel walls can be obtained in the following way (see [24]):

(14) $$<\rho \left(\tilde{x}\right)>=\frac{{\mathrm{IL}}_{\mu_{0}^{2}\to L_{0}}<\Psi \left(\tilde{x}\right)\cdot \vec{\phi }\left(x\right)\vec{\phi }\left(x^{\prime }\right){>}_{ww}}{{\mathrm{IL}}_{\mu_{0}^{2}\to L_{0}}<\vec{\phi }\left(x\right)\vec{\phi }\left(x^{\prime }\right){>}_{ww}}$$

in the limit n→0 . The average $<{>}_{ww}$ in Equation (14) denotes a statistical average for a Ginzburg–Landau field theory in the space between two walls. The dot in Equation (14) means the usual cumulant average, and IL is the inverse Laplace transform $\mu_{0}^{2}\to L_{0}$ .

Taking into account the normalization condition: $\int d^{d}\tilde{x}<\rho \left(\tilde{x}\right)>={\left(2R_{g}\right)}^{1/\nu }$ , the property:

(15) $$\int_{0}^{L}d\tilde{z}\int d^{d-1}\tilde{r}\mathrm{IL}<\Psi \left(\tilde{r},\tilde{z}\right)\cdot \vec{\phi }\left(x\right)\vec{\phi }\left(x^{\prime }\right){>}_{ww}={\left(2R_{g}\right)}^{1/\nu }\mathrm{IL}<\vec{\phi }\left(x\right)\vec{\phi }\left(x^{\prime }\right){>}_{ww},$$

takes place.

It should be noted that near the repulsive wall with the Dirichlet b.c., the short-distance expansion of $\Phi^{2}$ can be used [24,72,78,82], and it assumes:

(16) $$\Psi \left(\tilde{r},\tilde{z}\right)\to B{\tilde{z}}^{1/\nu }\frac{{\left[\Phi_{⊥}\left(\tilde{r}\right)\right]}^{2}}{2},$$

for the distances $l\ll \tilde{z}$ . The surface operator $\frac{{\left[\Phi_{⊥}\left(\tilde{r}\right)\right]}^{2}}{2}$ with $\Phi_{⊥}=\frac{\partial \Phi (\tilde{r},\tilde{z})}{\partial \tilde{z}}{|}_{\tilde{z}=0}$ is the component of the stress tensor perpendicular to the walls.

Taking into account the shift identity [73,78] in the case of two parallel walls situated at distance *L* from each other for the layer monomer density $\rho_{\lambda }\left(\tilde{z}\right)$ of the phantom ideal ring polymer in accordance with Equations (14) and (16), the universal density-force relation can be obtained for the region $l\ll \tilde{z}\ll R_{g}$ and can be written in a form similar to the case of linear polymer chains [24]:

(17) $$<\rho_{\lambda }\left(\tilde{z}\right)>=B{\tilde{z}}^{1/\nu }\frac{f}{k_{B}T},$$

where:

(18) $$\frac{f}{k_{B}T}=\lim_{\Delta x\to 0}\frac{d}{dL}\ln\left[\mathrm{IL}\int d^{d}x<\vec{\phi }\left(x\right)\vec{\phi }\left(x+\Delta x\right){>}_{ww}\right]$$

is the force per area that the polymer chain exerts on the walls. As was mentioned above, in the case of the ring polymer chain, during the calculations, we consider the limit Δx→0 . Accordingly, it is impossible to get the results for ring polymers directly from the results for linear polymer chains. Thus, this leads to the modification of the calculation scheme, which is connected with the reduction of some part of the integration during the calculation of the corresponding correlation function. The universal amplitude *B* is identified via scaling relations for the monomer density and force and is: $B=\lim_{x\to 0}x^{-1/\nu }X\left(x,y\right)/Y\left(y\right)$ , where *X* and *Y* are universal functions by analogy, as was proposed for linear polymer chains (see [24]).

**Figure 1:**
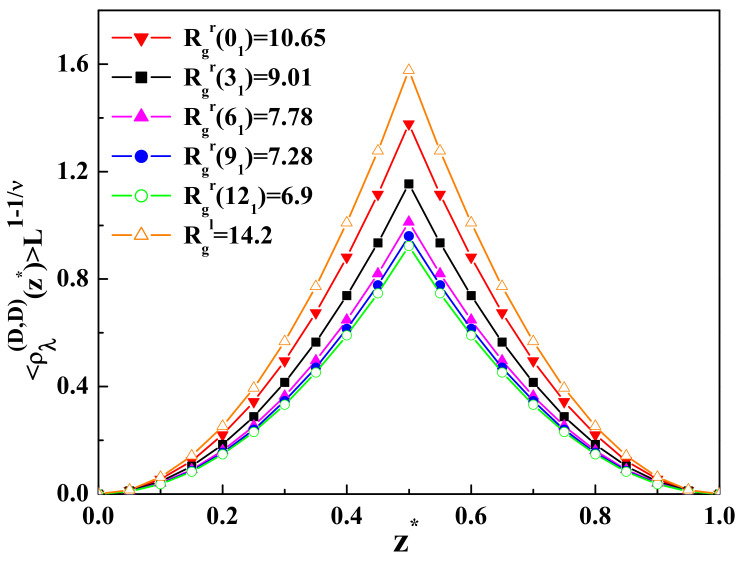
The dimensionless value of the layer monomer density $<\rho_{\lambda }^{\left(DD\right)}\left(z^{*}\right)>L^{1-1/\nu }$ profiles for the ideal ring polymer chain in a slit of two parallel walls with repulsive surfaces (Dirichlet-Dirichlet boundary conditions (D-D b.c.)) as a function of $z^{*}=\tilde{z}/L$ for different values of the radius of gyration. In the case of two repulsive surfaces, the maximum is at L/2 . The calculations are performed for L=25 .

After performing the Fourier transform in the direction parallel to the surfaces and integration over all possible positions of the ring polymer inside the slit with the D-D b.c. for the layer monomer density of the phantom ideal ring polymer, we obtained in the limit y≳1 (see Appendix C) the following result:

(19) $$\begin{array}{cc} <\rho_{\lambda }^{\left(DD\right)}\left(\tilde{z}\right)> & =\frac{{\left(2R_{g}\right)}^{1/\nu }}{L_{0}}\frac{\mathrm{IL}\int_{0}^{L}dzG\left(;z,\tilde{z}\right)G\left(;\tilde{z},z\right)}{\mathrm{IL}\int_{0}^{L}dzG\left(;z,z\right)}\\ \; & \approx 2\frac{{\tilde{z}}^{1/\nu }}{L}\left(1+\frac{\sqrt{\pi }}{2y}+\frac{\pi }{4y^{2}}+\frac{\pi^{3/2}}{8y^{3}}+O\left(e^{-4y^{2}}\right)\right), \end{array}$$

where $y=L/(2R_{g})$ . After taking into account the result for the reduced force per unit area, which the dilute polymer solution of ring polymers exerts on the walls in the limit y≳1 :

(20) $$\frac{f^{\left(DD\right)}}{k_{B}T}\approx \frac{1}{L}\left(1+\frac{\sqrt{\pi }}{2y}+\frac{\pi }{4y^{2}}+\frac{\pi^{3/2}}{8y^{3}}+O\left(e^{-4y^{2}}\right)\right)$$

and the result for the Flory exponent ν=1/2 at d=3 dimensions for ideal ring polymers, we come to the conclusion that the universal amplitude ratio *B* in the universal density-force relation in the case of phantom ideal ring polymer chains is equal to two and coincides with the result for the amplitude ratio in the case of the linear polymer chain [24,28,29]. Nevertheless, upon the different behavior of the layer monomer density profiles and forces for ring polymers (see Equations (19) and (20)) and for linear polymers [24,28,29], the universal amplitude ratio *B* in the density-force relation is the same. As one can see from Equation (19) in the limit y→∞ , the result for the layer monomer density of phantom ideal ring polymers in confined geometry coincides with the result for linear polymer chains (see [28,29]). The results of the calculations of the layer monomer density profiles for the case of the ideal ring polymer chain with different topological structures $0_{1},3_{1},6_{1},9_{1},12_{1}$ confined in a slit geometry of two parallel walls with repulsive surfaces (D-D b.c.), as well as one repulsive and one inert surface (D-N b.c.) are presented in Figure 1 and Table 1, respectively (see also Appendix C). These knots belong to the family of torus knots. $C_{p}$ is a standard notation [30], where *C* gives the minimum number of crossings in any projection onto a plane, the essential crossings. The index *p* is used to distinguish knots with equal *C*.

As it is possible to see from Figure 1, the more complicated topological structure results in reducing the layer monomer density profiles in the region between two repulsive surfaces. A similar behavior of the layer monomer densities is observed in the case of one repulsive and the other inert surface. As one can see from Table 1, the more complicated topological structure leads to reducing the layer monomer density profiles near the surface with the adsorption threshold (inert surface with the Neumann b.c.). The monomer density profiles of ring polymers with a complicated topological structure near adsorbing or inert surfaces behave in a different way than for the case of linear polymer chains. A complicated topological structure prevents the polymer from adsorption on the surface and leads to the decreasing of the monomer density in the region near the adsorbing surface or the surface with the adsorption threshold. As is possible to see from Table 1, the result for the monomer density as a function of $z^{*}$ for the ring polymer with the $0_{1}$ topology is smaller than the result for the linear chain and bigger than the results for ring polymers with the more complicated topology $3_{1},6_{1},9_{1},12_{1}$ . This means that due to the complicated topology of ring polymers and the presence of the topological constraints, a smaller number of monomers can be found near the adsorbing surface. It should be mentioned that in the case of two repulsive surfaces, the layer monomer density achieves the maximum in the middle of the slit, and in the case of one repulsive and the other one inert surface, the maximum is situated near the inert surface where the adsorption threshold takes place. The results presented in Figure 1 and Table 1 for the layer monomer density profiles of the ideal ring polymer chain of N=300 monomers with different topological structures $0_{1},3_{1},6_{1},9_{1},12_{1}$ are obtained for the respective values of the radius of gyration: $R_{g}^{r}\left(0_{1}\right)=10.65\pm 0.01\left(l\right)$ , $R_{g}^{r}\left(3_{1}\right)=9.01\pm 0.01\left(l\right)$ , $R_{g}^{r}\left(6_{1}\right)=7.78\pm 0.01\left(l\right)$ , $R_{g}^{r}\left(9_{1}\right)=7.28\pm 0.01\left(l\right)$ , and $R_{g}^{r}\left(12_{1}\right)=6.9\pm 0.01\left(l\right)$ , and for linear polymer chain with $R_{g}^{l}=14.2\pm 0.01$ . It should be mentioned that the above-mentioned results for the radius of gyration of the polymer chain with N=300 monomers were obtained in the framework of the bead-spring model using the molecular dynamic simulations by Matthews et al. in [40].

Taking into account the Derjaguin approximation [32], we performed the calculation of the layer monomer density profiles in the case when we have a dilute polymer solution of ring polymers in a solution of big colloidal particles with different adsorbing or repelling properties with respect to the polymers. In connection with this, we discus two cases of immersing a dilute polymer solution of ring polymers in confined geometries: (1) between a wall and a big colloidal particle; (2) between two big colloidal particles with different boundary conditions, D-D b.c. and D-N b.c.

The Derjaguin approximation [32], which describes the sphere by a superposition of fringes with the local distance from the wall $L\left(r_{\|}\right)=a+r_{\|}^{2}/\left(2R\right)$ , can be applied for the case of a spherical mesoscopic colloidal particle with radius *R* much larger than the distance from its closest point “*a*” to the surface and much larger than the radius of gyration $R_{g}$ of the ring polymer. Immersing the big spherical colloidal particle in the polymer solution confined in a semi-infinite space changes the force exerted on the wall by the value Δf . The depletion interaction of the particle with the wall can be obtained as the difference between the forces with and without the particle. The above-mentioned arguments and the density-force relation Equation (17) allow us to write the expression for the layer monomer density of a dilute polymer solution of ring polymers in a semi-infinite geometry containing a spherical particle of a big radius in the form:

(21) $$<\rho_{\lambda }\left(\tilde{z}\right){>}_{wp}=B{\tilde{z}}^{1/\nu }\left(\frac{\Delta f}{k_{B}T}+n_{B}\right),$$

by analogy, as was proposed for the case of linear polymer chains in [28,29]. Here, $n_{B}=\tilde{N}/V$ is the polymer density in the bulk far from the wall, and $\tilde{N}$ is the number of polymer chains in a solution. The depletion interaction potential for a dilute solution of ring polymers between the particle and the wall can be obtained according to [27,28]:

(22) $$\frac{\phi_{depl}\left(a\right)}{n_{B}k_{B}T}=8\pi RR_{g}^{2}\int_{\frac{a}{2R_{g}}}^{\infty }dy\Theta^{r,id}\left(y\right),$$

and it allows us to calculate the force $\Delta f=-d\phi_{depl}\left(a\right)/da$ . The $\Theta^{r,id}\left(y\right)$ in Equation (22) is the dimensionless scaling function of the free energy of a dilute solution of ring polymers confined in a slit geometry (see [65]). Taking into account that $\Theta^{r,id}\left(y\right)=\mp 2ye^{-2y^{2}}$ , where the sign “−” corresponds to D-D b.c. and “+” corresponds to D-N b.c., the layer monomer density of a dilute polymer solution of ring polymers in a semi-infinite space containing a spherical particle of a big radius can be obtained:

(23) $$<\rho_{\lambda }\left(\tilde{z}\right){>}_{r}=B\frac{{\tilde{z}}^{1/\nu }}{L}\left(1\mp 4a\pi \tilde{R}e^{\frac{-a^{2}}{2R_{g}^{2}}}\right),$$

where A=1 , $\tilde{R}=R$ , and $n_{B}=1/\left(LA\right)$ for the case of a dilute polymer solution of ring polymers between a wall and a big colloidal particle of radius *R*; $\tilde{R}=\frac{R_{1}R_{2}}{R_{1}+R_{2}}$ for the case of two colloidal particles of different sizes with radius $R_{1}\neq R_{2}$ . It should be mentioned that the sign “−” in Equation (23) refers to the case of two repulsive surfaces with the D-D b.c., and the sign “+” corresponds to the case of one repulsive and one inert surface with the D-N b.c.

We can see that the layer monomer density depends not only on $R_{g}$ , but also on the size of the mesoscopic particle and its distance from the wall. From Equation (23), we can see that in the case when we have two particles of the same size, the respective contribution to the layer monomer density profiles from immersing the particles becomes twice smaller than in the case when we have one particle near the wall.

**Figure 2:**
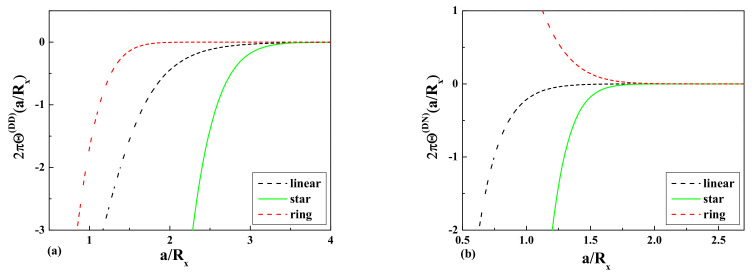
The dimensionless values of the contribution $2\pi \Theta (\frac{a}{R_{x}})$ to the normalized layer monomer density $\rho_{\lambda }\left(\tilde{z}\right)$ in the solution of big colloidal particles for the case of ideal linear, ring, and star polymers: (**a**) the case of the Dirichlet b.c.; (**b**) the case of the Neumann b.c.

We can compare the obtained results for the layer monomer density profiles of a dilute solution of ideal ring polymers confined in a solution of colloidal particles of big size with the results obtained for linear polymer chains obtained in [28]. In this respect, we should mention that in [28], there was a typographical error in Equations (2.28) and (2.29)) for the layer monomer density profiles $<\rho_{\lambda }\left(\tilde{z}\right){>}_{wp}$ of linear polymers in a semi-infinite space containing spherical particle of a big radius, but the final results presented in Figure 2a,b were correct (see [28]). For the case of a dilute solution of linear polymer chains in a semi-infinite space containing a mesoscopic spherical particle of a big size the monomer, the density profiles should be written in the form:

(24) $$<\rho_{\lambda }\left(\tilde{z}\right){>}_{wp}=B\frac{{\tilde{z}}^{1/\nu }}{L}\left(1+2\pi RR_{x}\Theta (\frac{a}{R_{x}})\right),$$

which is in agreement with our previous results for the dimensionless values of the depletion interaction potential and the depletion force, published recently in [31,50]. Here, the value $R_{x}$ corresponds to the value of the end-to-end distance for the linear polymer chain, and the values $\Theta (\frac{a}{R_{x}})$ for the dimensionless depletion interaction potential of ideal and real linear polymer chains with the excluded volume interaction (EVI) were calculated in [27]. Taking into account the above-mentioned arguments for the layer monomer density profiles of a dilute solution of ideal linear polymer chains confined in a solution of colloidal particles of a big size in accordance with [27] after the substitution of the expressions for $\Theta (\frac{a}{R_{x}})$ in (24), we obtain in the case of the D-D b.c.:

(25) $$\begin{array}{cc} <\rho_{\lambda }^{\left(DD\right)}\left(\tilde{z}\right){>}_{l}=B\frac{{\tilde{z}}^{1/\nu }}{L}(1 & +8a\pi \tilde{R}(\left[erfc\left[\frac{a}{\sqrt{2}R_{x}}\right]-\frac{R_{x}}{a}\sqrt{\frac{2}{\pi }}e^{-\frac{a^{2}}{2R_{x}^{2}}}\right]\;\;\\ \; & -\;\;2[erfc\left[\frac{\sqrt{2}a}{R_{x}}\right]-\frac{R_{x}}{a\sqrt{2\pi }}e^{\frac{-2a^{2}}{R_{x}^{2}}}])), \end{array}$$

and for the D-N b.c., the following expression takes place:

(26) $$<\rho_{\lambda }^{\left(DN\right)}\left(\tilde{z}\right){>}_{l}=B\frac{{\tilde{z}}^{1/\nu }}{l}\left(1+8a\pi \tilde{R}\left(erfc\left[\frac{\sqrt{2}a}{R_{x}}\right]-\frac{R_{x}}{a\sqrt{2\pi }}e^{-\frac{2a^{2}}{R_{x}^{2}}}\right)\right).$$

The comparison of the obtained results for $2\pi \Theta (\frac{a}{R_{x}})$ according to Equations (23)–(26) in the case of a dilute solution of ideal ring polymers and a solution of ideal linear polymers immersed in a solution of mesoscopic colloidal particles of a big size are presented in Figure 2a,b for the case of the D-D b.c. and the D-N b.c., respectively.

## 4. Small Sphere and Wall Interaction

Let us consider the opposite case of a small spherical colloidal particle (or nano-particle) with radius *R*, which is much smaller than the distance of the particle $z_{s}=L+\left(R/2\right)$ from the wall and much smaller than the polymer size $R_{x}$ (or $R_{g}$ for ring polymer chains). The effect of the presence of the particle on the polymer chain can be described in terms of a δ -function potential that weakly repels the monomers and is located at the center of the particle, as was proposed by Eisenriegler for the case of linear polymer chains (see [76]). It assumes that the Boltzmann weight $W_{s}$ for the chain that arises from the presence of the small sphere can be written in the form:

(27) $$W_{s}\left[x\right]\to 1-\tilde{A}R^{d-\frac{1}{\nu }}\rho \left(x_{s}\right),$$

where the amplitude $\tilde{A}$ is universal [25,83] and for the case of ideal polymer chain in *d* = 3 dimensions is: $\tilde{A}=2\pi$ . The modified monomer density ρ(x) can be written in the form [76]:

(28) $$\rho \left(x\right)=\sum_{k=1}^{\tilde{N}}\frac{R_{x}^{1/\nu }}{N}\sum_{j=1}^{N}\delta \left(x-x_{k,j}\right).$$

For free polymer chains in a half space, the following equation [76]:

(29) $$<\rho \left(x\right){>}_{\left(f,h\right)}=n_{B}R_{x}^{1/\nu }M\left(z/R_{x}\right),$$

where $M(z/R_{x})$ is the bulk normalized monomer density in the half space without the sphere. Taking into account the above-mentioned relation Equation (29), the universal relation in Equation (17) can be written in the form [24]:

(30) $$\frac{M(z/R_{x})}{z^{1/\nu }}=\frac{B}{R_{x}^{1/\nu }},$$

where we took into account that the pressure on the wall f/A according to the ideal gas law is equal to the pressure in the bulk: $n_{B}k_{B}T$ .

**Figure 3:**
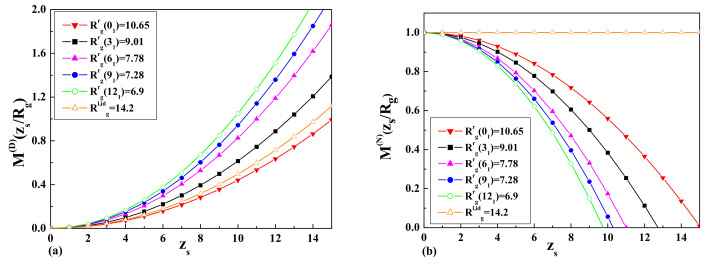
The dimensionless value of the normalized monomer density $M(z_{s}/R_{g})$ , where $R_{g}=R_{x}/2$ for ideal ring polymer chains: (**a**) the case of the Dirichlet b.c.; (**b**) the case of the Neumann b.c.

Immersing a small spherical particle with $R\ll z_{s},R_{x}$ in a polymer solution in the half space changes the polymer free energy per $k_{B}T$ by [76]:

(31) $$\frac{F}{k_{B}T}=-{\left\{W-1\right\}}_{\left(f,h\right)}=AR_{x}^{1/\nu }R^{d-(1/\nu )}n_{B}M\left(z_{s}/R_{x}\right),$$

where the small sphere expansion Equation (27) was taken into account. The results for the normalized monomer density $M(z_{s}/R_{g})$ , where $R_{g}=R_{x}/2$ for ideal ring polymer chains in the case of a wall with a surface at the Dirichlet b.c. (D b.c.) and a surface at the Neumann b.c. (N b.c.), are presented in Figure 3a,b, respectively.

The free energy of interaction δF between a small spherical particle and the wall follows from *F* on subtracting its value for infinite distance, i.e., at $z_{s}\to \infty$ (see [76]):

(32) $$\delta F=F-\lim_{z_{s}\to \infty }F.$$

Taking into account that $M(z_{s}\to \infty )=1$ for the free energy of interaction between the particle and the wall, the following equation can be obtained (see [76]):

(33) $$\delta F=-n_{B}k_{B}T\tilde{A}R_{x}^{1/\nu }R^{d-1/\nu }\left(1-M\left(z_{s}/R_{x}\right)\right).$$

Thus, the result for the force $\frac{\delta f}{n_{B}k_{B}T}$ , which arises in a dilute polymer solution between a small spherical particle with radius $R\ll z_{s},R_{x}$ and the wall, is:

(34) $$\delta f/\left(n_{B}k_{B}T\right)=-\partial_{z_{s}}\delta F=-\tilde{A}R_{x}^{1/\nu }R^{d-1/\nu }\partial_{z_{s}}M\left(z_{s}/R_{x}\right).$$

Taking into account the above-mentioned arguments by analogy with [76], we obtained the results for the force $\frac{\delta f}{n_{B}k_{B}T}$ at d=3 , which arises in a dilute solution of ideal linear polymer chains between a small spherical particle with radius $R\ll z_{s},R_{x}$ and the wall with a surface at the Neumann b.c. and a surface at the Dirichlet b.c., respectively:

(35) $$\frac{\delta f_{l}^{\left(N\right)}}{n_{B}k_{B}T}=0,\;\;\frac{\delta f_{l}^{\left(D\right)}}{n_{B}k_{B}T}=-8\pi Rz_{s}+O\left(z_{s}^{2}\right).$$

The results for the force in the case of a dilute solution of ring polymer chains at d=3 are the following:

(36) $$\frac{\delta f_{r}^{\left(N\right)}}{n_{B}k_{B}T}=8\pi Rz_{s}+O\left(z_{s}^{2}\right),\;\;\frac{\delta f_{r}^{\left(D\right)}}{n_{B}k_{B}T}=-8\pi Rz_{s}+O\left(z_{s}^{2}\right).$$

It should be mentioned that the result in Equation (35) for the force in the case of a small colloidal particle (or nano-particle) near the wall with the surface at the Neumann b.c. indicates that a dilute polymer solution of ideal linear polymer chains behaves in such a way that it does not feel this small particle. This result is in agreement with our previous result for a dilute solution of linear polymer chains in a slit of two parallel walls with both inert surfaces at the N-N b.c. [27] and the result for the case of a dilute solution of ideal linear polymer chains in a solution of big colloidal particles with the D-D b.c. [31]. As it is possible to see from the obtained results (see Equations (35) and (36)), in the case of a dilute solution of linear or ring polymers immersed between a small colloidal particle (or nano-particle) with radius $R\ll z_{s},R_{x}$ and the wall with the surface at the Dirichlet b.c., the respective depletion force is attractive. This result assumes that polymer chains tend to escape from the space between a small particle and a wall with the surface at the Dirichlet b.c., and this leads to an unbalanced pressure from the outside and the attraction between a small colloidal particle (or nano-particle) and the wall. In the case when we consider a dilute solution of ideal ring polymer chains confined in a solution of small colloidal particles (or nano-particles) near the wall with the surface at the Neumann b.c., we observe that the polymer adsorbs on the wall, and this leads to the repulsion between small particles and the wall. This result is in agreement with our previous predictions for a dilute polymer solution of ring polymers in a slit geometry of two parallel mixed walls with one surface at the Neumann b.c. and the other one at the Dirichlet b.c. (see [65]), as well as with the result for a dilute solution of ideal ring polymer chains in a solution of big colloidal particles with mixed boundary conditions (D-N b.c.) [31,50].

## 5. Star Polymers

In the case of a dilute solution of star polymers with the number of f=4 arms in a Θ -solvent immersed in a confined geometry like a slit of two parallel walls, the respective correlation function $G_{st}^{f}\left(\mu_{0}\right)$ should be modified and can be written in the form:

(37) $$G_{st}^{f}\left(\mu_{0}\right)=<\sum_{j_{1},\ldots ,j_{f}=1}^{n}T_{i_{1},\ldots ,i_{f}}\phi^{i_{1}}\left(x_{0}\right)\ldots \phi^{i_{f}}\left(x_{0}\right)\phi^{j_{1}}\left(x_{1}\right)\ldots \phi^{j_{f}}\left(x_{f}\right){>}_{n\to 0}^{H_{st}},$$

where the Hamiltonian $H_{st}\left[\vec{\phi }\right]$ has a form:

$$\begin{array}{cc} H_{st}\left[\vec{\phi }\right] & =\sum_{a=1}^{f}\int d^{d}x\left\{\frac{1}{2}{\left(\nabla {\vec{\phi }}_{a}\right)}^{2}+\frac{\mu_{0,a}^{2}}{2}{{\vec{\phi }}_{a}}^{2}\right\}\\ & +\sum_{a=1}^{f}\sum_{j=1}^{2}\frac{c_{j_{0},a}}{2}\int d^{d-1}r{{\vec{\phi }}_{a}}^{2}. \end{array}$$

We consider a dilute solution of star polymers with four arms immersed into a slit geometry of two parallel walls, and in the field theory, the star vertex is related to the local composite operator (see [84]) appearing in Equation (37):

(38) $${\left(\phi \right)}_{st}^{f}\left(x\right)=T_{i_{1},\ldots ,i_{f}}\phi^{i_{1}}\left(x\right)\ldots \phi^{i_{f}}\left(x\right),$$

where $T_{i_{1},\ldots ,i_{f}}$ is a traceless symmetric SO (n) tensor fulfilling the condition:

(39) $$\sum_{i=1}^{m}T_{i,i,i_{3},\ldots ,i_{f}}=0.$$

Taking into account the above-mentioned arguments of the calculation of the free energy of the system, the respective partition function $Z_{\|,st}^{f}$ of the star polymer of f=4 arms immersed in a slit geometry of two parallel walls at a distance *L* should be normalized on the partition function $Z_{st}$ of one star polymer in the same volume *V* without walls. Following the scheme, proposed in [27] for the case of a dilute solution of linear polymers, we can obtain the results for the dimensionless depletion interaction potential:

(40) $$\Theta {\left(y\right)}_{st}^{\left(DD\right)}=-\left(\frac{61}{7}+\frac{1584}{175}y^{2}\right)\sqrt{\frac{5}{\pi }}e^{-\frac{4y^{2}}{5}}+\left(\frac{155}{7}+\frac{10,272}{175}y^{2}\right)\sqrt{\frac{5}{\pi }}e^{-\frac{16y^{2}}{5}}+\ldots ,$$

as well as the dimensionless depletion force:

(41) $$\Gamma {\left(y\right)}_{st}^{\left(DD\right)}=\left(\frac{91}{5}-\frac{1584}{25}y^{2}\right)\sqrt{\frac{5}{\pi }}\frac{8y}{35}e^{-\frac{4}{5}y^{2}}+\left(\frac{133}{5}+\frac{12,672}{25}y^{2}\right)\sqrt{\frac{5}{\pi }}\frac{32y}{35}e^{-\frac{16}{5}y^{2}}+\ldots ,$$

in the case of a dilute solution of star polymers with four arms in a slit of two parallel walls with the D-D b.c. In the case of two parallel walls with one repulsive and the other inert surface, which corresponds to the D-N b.c., we obtain respectively:

(42) $$\Theta {\left(y\right)}_{st}^{\left(DN\right)}=-\left(\frac{61}{14}+\frac{3168}{175}y^{2}\right)\sqrt{\frac{5}{\pi }}e^{-\frac{16}{5}y^{2}}+\ldots ,$$

and:

(43) $$\Gamma {\left(y\right)}_{st}^{\left(DN\right)}=\left(\frac{208}{5}-\frac{101,376y^{2}}{175}\right)\sqrt{\frac{5}{\pi }}\frac{y}{5}e^{-\frac{16y^{2}}{5}}+\ldots .$$

**Figure 4:**
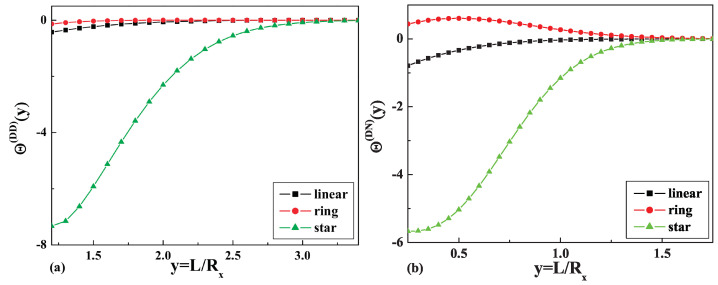
The dimensionless values of the depletion interaction potentials: (**a**) $\Theta {\left(y\right)}^{\left(DD\right)}$ and (**b**) $\Theta {\left(y\right)}^{\left(DN\right)}$ for the case of a linear, a ring, and a star polymer with four arms.

**Figure 5:**
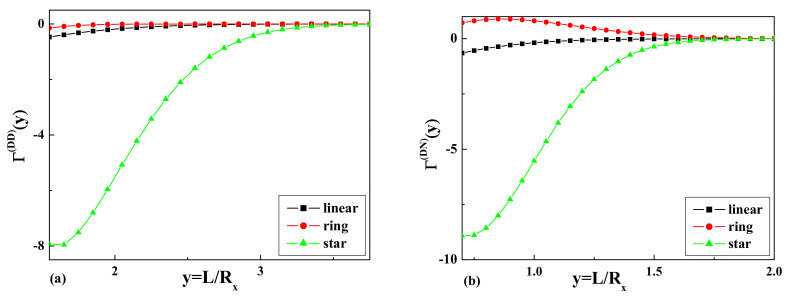
The dimensionless values of the dimensionless depletion forces: (**a**) $\Gamma {\left(y\right)}^{\left(DD\right)}$ and (**b**) $\Gamma {\left(y\right)}^{\left(DN\right)}$ for the case of a linear, a ring, and a star polymer with four arms.

The results of the calculations of the dimensionless depletion interaction potentials $\Theta {\left(y\right)}_{st}^{\left(DD\right)}$ and $\Theta {\left(y\right)}_{st}^{\left(DN\right)}$ , as well as the dimensionless depletion forces $\Gamma {\left(y\right)}_{st}^{\left(DD\right)}$ and $\Gamma {\left(y\right)}_{st}^{\left(DN\right)}$ and the comparison with the corresponding values for the linear and ring polymers are presented in Figure 4 and Figure 5, respectively.

Taking into account the Derjaguin approximation [32], the monomer density profiles of a dilute polymer solution of star polymers immersed between a wall and a big colloidal particle or in the case of two big colloidal particles of different sizes with different adsorbing and repelling properties with respect to polymers can be obtained. For example, in the case when both surfaces are repulsive for star polymers (the D-D b.c. case), we obtain:

(44) $$\begin{array}{cc} <\rho_{\lambda }^{\left(DD\right)}\left(\tilde{z}\right){>}_{st}=B\frac{{\tilde{z}}^{1/\nu }}{L}(1 & -2\sqrt{5\pi }\tilde{R}a[\left(\frac{61R_{x}}{7a}+\frac{1584}{175}\frac{a}{R_{x}}\right)e^{-\frac{4a^{2}}{5R_{x}^{2}}}\\ \; & -\left(\frac{155R_{x}}{7a}+\frac{10,272}{175}\frac{a}{R_{x}}\right)e^{-\frac{16a^{2}}{5R_{x}^{2}}}]), \end{array}$$

and in the case when one surface is at the adsorption threshold and the other one is repulsive, which corresponds to the case of the D-N b.c., we have:

(45) $$<\rho_{\lambda }^{\left(DN\right)}\left(\tilde{z}\right){>}_{st}=B\frac{{\tilde{z}}^{1/\nu }}{L}\left(1-2\sqrt{5\pi }\tilde{R}a\left[\left(\frac{61R_{x}}{14a}+\frac{3168}{175}\frac{a}{R_{x}}\right)e^{-\frac{16a^{2}}{5R_{x}^{2}}}\right]\right).$$

The comparison of the results Equations (44) and (45) obtained for the layer monomer density profiles of a dilute solution of star polymers with f=4 arms with the results obtained for ring polymers Equation (23) and linear polymers Equations (25) and (26) immersed in a solution of mesoscopic colloidal particles of a big size with different adsorbing and repelling properties with respect to polymers are presented in Figure 2a,b, respectively.

## 6. Molecular Dynamic Simulations of Linear, Ring, and Star-Shaped Polymers in a Slit

**Figure 6:**
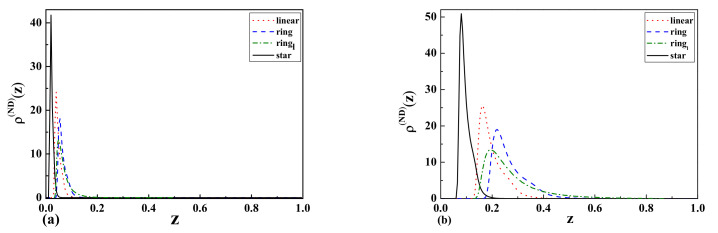
The monomer density profiles ρ(z) of linear, ring, and star-shaped polymers between attractive and repulsive walls corresponding to Neumann-Dirichlet (ND) b.c. with the separation of (**a**) $L=2R_{g}$ (wide slit) and (**b**) $L=R_{g}/2$ (narrow slit).

We created a computer program in C++ for the simulations of polymers in a slit. We performed molecular dynamics simulations of a dilute solution of linear, ring, and star-shaped polymers consisting of N=300 , 300, and 1201 (4 × 300+1-star with four arms) monomers accordingly. The starting configuration was chosen in such a way, that monomers were placed on the ideal line, ring, or star. The interaction of the neighboring monomers was modeled by the finite extensible nonlinear elastic (FENE) (attractive part) and the Weeks-Chandler-Andersen (WCA) (repulsive part) potentials. The selected potential, which tends to infinity as R→∞ and has one minimum, ensures that all selected topologies are preserved during the simulations. Additionally, we take into account the long-range interactions (the 12-6 Lennard–Jones potential) between monomers along the chain. Before the data acquisition, we equilibrated the whole system. We used the Verlet integration scheme with Δt=0.005 and the velocity scaling thermostat, which helped to keep the temperature T=1 constant. All simulations were equilibrated for $t_{eq}=500$ , followed by data collection for $t_{data}$ from 500 to 3500. First, to establish the radius of gyration $R_{g}$ , we performed 10 simulations for each polymer shape. We obtained $R_{g}=14.65\left(47\right)$ for linear, $R_{g}=10.91\left(15\right)$ for ring, and $R_{g}=29.43\left(68\right)$ for star polymers. Then, we performed simulations for polymers between two walls with *L* separation, which was set to either $2R_{g}$ or $R_{g}/2$ . The interaction of the monomers with the walls was modeled by the 9-3 Lennard–Jones potential with a variable cut-off. The potential cut-off was set to 1.2 (repulsive walls) or 10 (attractive walls). Depending on the separation of the walls and their type, the monomer density profiles were calculated as average values of up to 10 independent simulations. The separations of the walls were normalized to one. All the monomer densities were then normalized as: $\int_{0}^{1}\rho \left(z\right)dz=\frac{R_{g}}{L}$ to compare the results regardless of the length of the polymers. Additionally, we performed similar molecular dynamics simulations using LAMMPS (Large-scale Atomic/Molecular Massively Parallel Simulator) for ring polymers consisting of 360 beads. The simulation model was set up similarly to our own program. The monomer densities were taken from 10 separate simulations. The results of the simulations can be seen in Figure 6a,b denoted as $ring_{l}$ in the legend. The results obtained with both numerical methods (our program and LAMMPS), in spite of different numbers of monomers, represent the same qualitative behavior.

**Figure 7:**
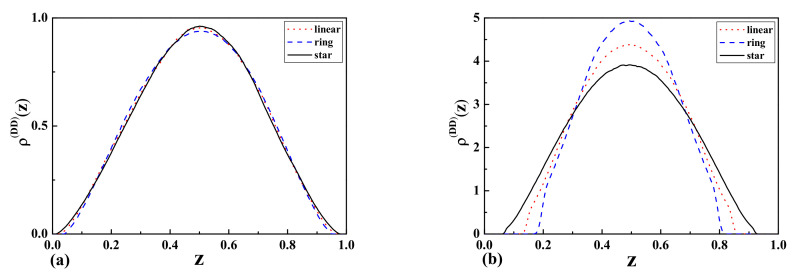
The monomer density profiles ρ(z) of linear, ring, and star-shaped polymers between two repulsive walls corresponding to Dirichlet-Dirichlet (DD) b.c. with the separation of (**a**) $L=2R_{g}$ (wide slit) and (**b**) $L=R_{g}/2$ (narrow slit).

For the case of two repulsive walls with separation $L=2R_{g}$ (wide slit), we observed that the monomer density profiles for star polymers are higher than the results for linear and ring polymers in the middle of the slit and near the walls (Figure 7a). As one can see from Figure 7b, the situation looks completely different in the case of a narrow slit. In this case, the monomer density profiles for ring polymers are higher than the results for linear and star polymers in the middle of the slit. This can be attributed to the different topologies of polymers, as well as their corresponding $R_{g}$ values. It should be mentioned that the behavior of the profiles near the walls is opposite.

In the case of one attractive and one repulsive wall (Figure 7a,b, we observed that the monomer density profiles for star polymers are higher than the results for linear and ring polymers. Furthermore, the maxima of peaks for the above-mentioned cases are shifted for wide and narrow slits. We observed in the case of wide-slit polymers that they are not influenced by the presence of the repulsive wall. The situation is different in the case of the narrow slit, where the positions and shapes of peaks are shifted when compared to the wide slit.

**Figure 8:**
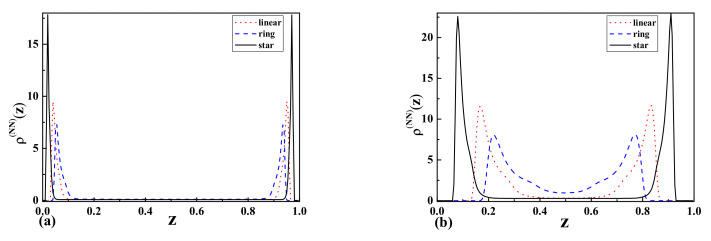
The monomer density profiles ρ(z) of linear, ring, $ring_{l}$ , and star-shaped polymers between two attractive walls corresponding to Neumann-Neumann (NN) b.c. with the separation of (**a**) $L=2R_{g}$ (wide slit) and (**b**) $L=R_{g}/2$ (narrow slit).

Figure 8a,b presents the result for two attractive walls. The resulting monomer density profiles indicate that the polymer tends to stay near the attractive walls in a similar way as in the previous case for the wide slit region. The biggest difference is observed in the case of the narrow slit where a non-zero monomer density is observed in the middle of the slit, especially for ring polymers.

As one can see from Figure 6a, Figure 7a,b Figure 8b, the topological and entropic effects play a crucial role in the monomer density profiles near the walls. The obtained numerical results (see Figure 6a and Figure 7a) for two repulsive walls, as well as for the case of one attractive and the other repulsive wall are in good qualitative agreement with our analytical results presented in Figure 1 and Table 1 for the above-mentioned cases.

## 7. Summary

In the present paper, the investigation of a dilute solution of ideal ring polymer chains, as well as a dilute solution of star polymers with f=4 arms in a Θ -solvent immersed in confined geometries like the slit of two parallel walls and in a solution of big and small spherical colloidal particles with different adsorbing and repelling properties with respect to polymers was performed.

The layer monomer density profiles $<\rho_{\lambda }\left(\tilde{z}\right)>$ of a dilute solution of ideal ring polymer chains immersed in a slit geometry of two parallel walls with repulsive surfaces, as well as in the case of one repulsive and the other inert surface were obtained. It should be mentioned that in the case of two repulsive surfaces, the maximum of the layer monomer density is in the middle of the slit at L/2 . In the case of one repulsive and the other inert surface, the maximum of the layer monomer density is near the distant wall with the inert surface, where the adsorption threshold takes place. As one can see from Equations (19), (A6), and (A9), Figure 1, and Table 1, the monomer density profiles of ring polymers depend on the value $y=L/(2R_{g})$ . The smaller radius of gyration corresponds to the more complicated topological structure. It leads to the decreasing of the layer monomer density with increasing the complexity of the topological structure in the region between two repulsive surfaces, as well as in the region near the surface where the adsorption threshold takes place in the case of mixed surfaces. We have come to the conclusion that the complicated topological structure of ring polymers prevents them from adsorption on the surface and leads to the decreasing of the layer monomer density in the region near the adsorbing surface.

We obtained (see Equations (19), (20), (25), and (26)) that regardless of the different behaviors of the layer monomer density profiles and forces for ring polymers and for linear polymers, the universal amplitude ratio *B* in the density-force relation is the same as for linear polymer chains and equals two. Taking into consideration the Derjaguin approximation, the layer monomer density profiles of a dilute polymer solution of ring polymers confined in a semi-infinite space containing the mesoscopic spherical particle of a big radius *R* and two big spherical particles (such that $R_{i}\gg R_{g}$ and $R_{i}\gg a$ with i=1,2 ) for the case of the D-N b.c. and D-D b.c. were obtained. We can see that the layer monomer density depends on the radius of gyration of ring polymers, the size of the mesoscopic particle, and the distance between the wall and particle or between two particles.

We obtained that the relation between the monomer density profiles of ring polymer chains with knot type $0_{1}$ to the monomer density profiles of ideal linear polymer chains in the case of two parallel walls with repulsive surfaces, as well as with one repulsive and the other inert surface is equal to one. Our analytical results are in good qualitative agreement with the previous predictions obtained for a bead-spring model using molecular dynamic simulations by Matthews et al. [40] for two repulsive surfaces, as well as with our numerical results presented in Figure 6a and Figure 7a for the case of two repulsive walls and one attractive and the other repulsive wall. We come to the conclusion that topological and entropic effects play a crucial role in the monomer density profiles in confined geometries.

In the case when we consider a dilute solution of ring polymers at Θ -temperature immersed in a solution of small colloidal particles and the wall with the surface at the Neumann b.c., we observed that polymers adsorb on the wall. The results in the case of a dilute solution of ring or linear polymers at the Dirichlet b.c. show that polymer chains tend to escape from the space between the wall and a small particle or nano-particle.

We investigated a dilute solution of linear and ring polymers at Θ -temperature immersed in a solution of small colloidal particles. In the case of a small colloidal particle (or nano-particle) near the wall with the surface at the Neumann b.c., we obtain that the force is equal to zero for a dilute solution of linear polymer chains, and this indicates that a dilute polymer solution is not influenced by the presence of the wall. In the case when we consider a dilute solution of ideal ring polymer chains confined in a solution of small colloidal particles (or nano-particles) near the wall with the surface at the Neumann b.c., we observe that polymer adsorbs on the wall, and this leads to the repulsion between small particles and the wall. The above-mentioned result is in agreement with the result for a dilute solution of ideal ring polymer chains in a solution of big colloidal particles with mixed boundary conditions (D-N b.c.) [31,50].

As one can see from the obtained results (see Equations (35) and (36)), in the case of a dilute solution of linear or ring polymers immersed in a solution of small colloidal particles (or nano-particles) with radius $R\ll z_{s},R_{x}$ and the wall with the surface at the Dirichlet b.c., the respective depletion force becomes attractive. This result assumes that linear, as well as ring polymers tend to escape from the space between a small particle and the wall with the surface at the Dirichlet b.c., and this leads to an unbalanced pressure from the outside and the attraction between a small colloidal particle (or nano-particle) and the wall.

We obtained the dimensionless depletion interaction potentials, as well as the dimensionless depletion forces for the case of a dilute solution of star polymers with f=4 arms immersed in a slit geometry of two parallel walls with repulsive surfaces and with one repulsive and the other inert surface. The obtained results (see Figure 5a,b) indicate that the depletion force in both cases for a dilute solution of star polymers is attractive, but bigger than the respective forces for linear and ring polymer chains in the case of two repulsive surfaces. It should be mentioned that the depletion force in the case of one repulsive and the other inert surface is smaller than in the case of two repulsive surfaces and demonstrates the opposite behavior in the case of a dilute solution of ring polymer chains in confined geometries of two parallel walls with mixed surfaces.

The obtained results indicate that a dilute solution of ring and star polymer chains can be used for the production of new functional materials because the behavior of these solutions depends on the topology of polymers, as well as on the nature and geometry of confined surfaces. These properties can find wide application in nanotechnology, as well as in biotechnology for drug and gene transmission. We observe that in a narrow slit, ring polymers behave similarly to linear polymers, and this allows us to understand the process of the transmission of DNA from λ -bacteriophages to *E. coli* bacterial cells. In a wide slit region, the behavior of ring polymers depends on the complexity of the topological structure, and in the case of mixed walls with different adsorbing or repelling properties with respect to polymers, we observe that such polymers start to adsorb on the attractive surface; this leads to the repulsive forces and, as a consequence, to the destruction of the bacterial cell.

## Figures and Tables

**Table 1:** The dimensionless value of the layer monomer density $<\rho_{\lambda }^{\left(DN\right)}\left(z^{*}\right)>L^{1-1/\nu }$ profiles for the ideal ring polymer chain confined in a slit geometry of two parallel walls with one repulsive and the other inert surface (Dirichlet–Neumann boundary conditions (D-N b.c.)) as a function of $z^{*}=\tilde{z}/L$ (see Appendix B). The calculations are performed for L=20 .

$z^{*}$	0.0	0.2	0.4	0.6	0.8	1.0
$R_{g}^{l}$	0.0	0.09252	0.37007	0.83267	1.48030	2.31296
$R_{g}^{r}\left(0_{1}\right)$	0.0	0.08709	0.33417	0.74126	1.30834	2.03543
$R_{g}^{r}\left(3_{1}\right)$	0.0	0.08119	0.32239	0.72358	1.28477	2.00597
$R_{g}^{r}\left(6_{1}\right)$	0.0	0.08016	0.32033	0.72049	1.28065	2.00082
$R_{g}^{r}\left(9_{1}\right)$	0.0	0.08006	0.32011	0.72017	1.28022	2.00028
$R_{g}^{r}\left(12_{1}\right)$	0.0	0.08002	0.32004	0.72006	1.28009	2.00011

## Data Availability

Data sharing not applicable.

## Appendix A

As is well known [85], in the continuous chain limit l→0 , N→∞ with fixed $L_{0}=Nl^{2}$ , the partition function in (2) can be expressed as:

(A1)
$$Z_{c}\left(x^{\prime },L_{0}|x,0\right)=\Sigma_{\alpha }e^{-E_{\alpha }L_{0}}\phi_{\alpha }^{*}\left(x^{\prime }\right)\phi_{\alpha }\left(x\right),$$

where the $\phi_{\alpha }$ are a complete set of orthonormalized eigenfunctions (i.e., $<\phi_{\alpha }|\phi_{\alpha^{\prime }}>=\delta_{\alpha \alpha^{\prime }}$ of a Schrödinger equation:

(A2)
$$\left[-\Delta +U\left(x\right)\right]\phi_{\alpha }\left(x\right)=E_{\alpha }\phi_{\alpha }.$$

In accordance with $\Sigma_{\alpha }\phi_{\alpha }^{*}\left(x^{\prime }\right)\phi_{\alpha }\left(x\right)=\delta \left(x^{\prime }-x\right)$ in the case $L_{0}\to 0$ , $Z_{c}\left(x^{\prime },L_{0}|x,0\right)=\delta \left(x^{\prime }-x\right)$ takes place. The eigenfunctions extend much farther from the wall than the potential *U*. The partition function in Equation (2) fulfills boundary condition (3). As was shown in [15,16,17], the continuous chain model in Equation (2) can be mapped onto a corresponding field theory by a Laplace transform in the Gaussian variables $L_{0}$ to conjugate chemical potentials $\mu_{0}$ :

(A3)
$$G^{\left(2\right)}\left(x,x^{\prime }\right)=\int_{0}^{\infty }dL_{0}e^{-\mu_{0}L_{0}}Z_{c}\left(x^{\prime },L_{0}|x,0\right)=\Sigma_{\alpha }{\left(\mu_{0}+E_{\alpha }\right)}^{-1}\phi_{\alpha }^{*}\left(x^{\prime }\right)\phi_{\alpha }\left(x\right).$$

One the other hand, the Laplace transformed function $G^{\left(2\right)}\left(x,x^{\prime }\right)$ can be expressed as the n→0 limit of the functional integral over vector fields $\vec{\phi }\left(x\right)$ with *n* components $\phi_{i}\left(x\right)$ , i=1,…,n and x=(r,z) :

(A4)
$$G^{\left(2\right)}\left(x,x^{\prime }\right)=\int D\left[\vec{\phi }\left(x\right)\right]e^{-H[\vec{\phi }]}$$

with the Landau–Ginzburg–Wilson Hamiltonian $H(\vec{\phi })$ describing the system in the semi-infinite ( j=1 ) or confined geometry of two parallel walls ( j=1,2 ).

In our calculations, we use the polymer-magnet analogy developed by de Gennes [15,16,17], which means that we concentrate on the calculations of the correlation function $G^{\left(2\right)}\left(x,x^{\prime }\right)$ according to Equation (5) with the LGW Hamiltonian by analogy, as was proposed in [27].

In our calculations, we use the polymer-magnet analogy developed by de Gennes [15,16,17] and by Barber et al. [70] for the investigation of the critical behavior of polymers near the surface and in the case of slit geometry. This means that we concentrate on the calculations of the correlation function $G^{\left(2\right)}\left(x,x^{\prime }\right)$ according to Equation (5) with the Landau–Ginzburg–Wilson Hamiltonian by analogy, as was proposed in [27].

## Appendix B

The free propagator of the model (6) obtained in [27] is:

(A5)
$$\begin{array}{cc} {\tilde{G}}_{\|}\left(p;z,z^{\prime };\mu_{0},c_{1_{0}},c_{2_{0}},L\right) & =\frac{1}{2\kappa_{0}}(\left(\kappa_{0}^{2}+\kappa_{0}\left(c_{1_{0}}+c_{2_{0}}\right)+c_{1_{0}}c_{2_{0}}\right)e^{\kappa_{0}L}\\ & -\left(\kappa_{0}^{2}-\kappa_{0}\left(c_{1_{0}}+c_{2_{0}}\right)+c_{1_{0}}c_{2_{0}}\right)e^{-\kappa_{0}L}{)}^{-1}\\ & (\left(\kappa_{0}^{2}+\kappa_{0}\left(c_{1_{0}}+c_{2_{0}}\right)+c_{1_{0}}c_{2_{0}}\right)e^{\kappa_{0}(L-|z-z^{\prime }\left|\right)}\\ & +\left(\kappa_{0}^{2}-\kappa_{0}\left(c_{1_{0}}+c_{2_{0}}\right)+c_{1_{0}}c_{2_{0}}\right)e^{-\kappa_{0}(L-|z-z^{\prime }\left|\right)}\\ & +\left(\kappa_{0}^{2}+\kappa_{0}\left(c_{2_{0}}-c_{1_{0}}\right)-c_{1_{0}}c_{2_{0}}\right)e^{\kappa_{0}\left(L-z-z^{\prime }\right)}\\ & +\left(\kappa_{0}^{2}-\kappa_{0}\left(c_{2_{0}}-c_{1_{0}}\right)-c_{1_{0}}c_{2_{0}}\right)e^{-\kappa_{0}\left(L-z-z^{\prime }\right)}), \end{array}$$

with $\kappa_{0}=\sqrt{p^{2}+\mu_{0}^{2}}$ , where p is the value of the parallel momentum associated with d−1 translationally invariant directions in the system.

In the case when L→∞ and 0≤z≪L (or 0≪z≤L ), the free propagator in Equation (A5)) reproduces the correspondent free propagator of the semi-infinite model (see [27,77]) with the surface situated at z=0 and the surface enhancement constant $c_{1_{0}}$ or at z=L with $c_{2_{0}}$ . Thus, for infinitely large wall separations, the slit system decomposes into two half-space systems [27].

## Appendix C

After performing the Fourier transform in the direction parallel to the surfaces and integrated with all possible positions of the ring polymer inside the slit with the D-D b.c. for the layer monomer density of the phantom ideal ring polymer, we obtain:

(A6)
$$\begin{array}{cc} <\rho_{\lambda }^{\left(DD\right)}\left(\tilde{z}\right)> & =\frac{{\left(2R_{g}\right)}^{1/\nu }}{L_{0}}\frac{\mathrm{IL}\int_{0}^{L}dzG\left(;z,\tilde{z}\right)G\left(;\tilde{z},z\right)}{\mathrm{IL}\int_{0}^{L}dzG\left(;z,z\right)}\\ & \approx 2\frac{{\tilde{z}}^{1/\nu }}{L}\frac{\left(1+2e^{-4y^{2}}+2e^{-16y^{2}}\right)}{\left(1+2e^{-4y^{2}}+2e^{-16y^{2}}\right)-\frac{\sqrt{\pi }}{2y}}+O\left(e^{-4y^{2}}\right)\;\;\; \end{array}$$

Taking into account that we consider the d=3 dimensional case and in the wide slit limit with y≳1 , from Equation (A6), we obtain the result presented in Equation (19). For the sake of convenience, the result in Equations (A6) and (19) at d=3 dimensions with ν=1/2 can be written in the form:

(A7)
$$<\rho_{\lambda }^{\left(DD\right)}\left(z^{*}\right)>L^{-1}\approx 2{\left(z^{*}\right)}^{2}\left(1+\frac{\sqrt{\pi }}{2y}+\frac{\pi }{4y^{2}}+\frac{\pi^{3/2}}{8y^{3}}+O\left(e^{-4y^{2}}\right)+\ldots \right),$$

where $z^{*}=\frac{\tilde{z}}{L}$ .

The corresponding result for the layer monomer density of the phantom ideal ring polymer inside the slit with the D-N b.c. is:

(A8)
$$\begin{array}{cc} <\rho_{\lambda }^{\left(DN\right)}\left(\tilde{z}\right)> & =\frac{{\left(2R_{g}\right)}^{1/\nu }}{L_{0}}\frac{\mathrm{IL}\int_{0}^{L}dzG\left(;z,\tilde{z}\right)G\left(;\tilde{z},z\right)}{\mathrm{IL}\int_{0}^{L}dzG\left(;z,z\right)}\\ & \approx 2\frac{{\tilde{z}}^{1/\nu }}{L}+\frac{\tilde{z}}{y^{2}}\frac{\left(e^{-4y^{2}}-e^{-16y^{2}}\right)}{\left(1-2e^{-4y^{2}}+2e^{-16y^{2}}\right)}, \end{array}$$

which in the limit y≳1 at d=3 dimensions and with ν=1/2 leads to the result:

(A9)
$$<\rho_{\lambda }^{\left(DN\right)}\left(z^{*}\right)>L^{-1}\approx 2{\left(z^{*}\right)}^{2}+\frac{z^{*}}{y^{2}}\left(e^{-4y^{2}}+2e^{-8y^{2}}+4e^{-12y^{2}}-e^{-16y^{2}}+\ldots \right).$$
