# Correction: Halun et al. Investigation of Ring and Star Polymers in Confined Geometries: Theory and Simulations. *Entropy* 2021, *23*, 242

**DOI:** 10.3390/e24030413

**Published:** 2022-03-16

**Authors:** Joanna Halun, Pawel Karbowniczek, Piotr Kuterba, Zoriana Danel

**Affiliations:** 1 Institute of Nuclear Physics, Polish Academy of Sciences, 31-342 Cracow, Poland; 2 Institute of Physics, Cracow University of Technology, 30-084 Cracow, Poland; pkarbowniczek@pk.edu.pl (P.K.); zoriana.danel@pk.edu.pl (Z.D.); 3 Faculty of Physics, Astronomy and Applied Computer Sciences, Jagiellonian University in Cracow, 30-348 Cracow, Poland; piotr.kuterba@uj.edu.pl

The authors wish to make the following correction to this paper [1]. In our paper [1] are a few errors:

1. On page 8 symbol ‘cc’ before $<\rho_{\lambda }^{\left(DD\right)}\left(\tilde{z}\right)>$ in the Equation (19) should be removed and looks like:
  (19) $$\begin{array}{cc} <\rho_{\lambda }^{\left(DD\right)}\left(\tilde{z}\right)> & =\frac{{\left(2R_{g}\right)}^{1/\nu }}{L_{0}}\frac{\mathrm{IL}\int_{0}^{L}dzG\left(;z,\tilde{z}\right)G\left(;\tilde{z},z\right)}{\mathrm{IL}\int_{0}^{L}dzG\left(;z,z\right)}\\ \; & \approx 2\frac{{\tilde{z}}^{1/\nu }}{L}\left(1+\frac{\sqrt{\pi }}{2y}+\frac{\pi }{4y^{2}}+\frac{\pi^{3/2}}{8y^{3}}+O\left(e^{-4y^{2}}\right)\right) \end{array}$$
2. On page 12 symbol ‘cc’ before $<\rho_{\lambda }^{\left(DD\right)}\left(\tilde{z}\right){>}_{l}$ in the Equation (25) should be removed and looks like:
  (25) $$\begin{array}{cc} <\rho_{\lambda }^{\left(DD\right)}\left(\tilde{z}\right){>}_{l}=B\frac{{\tilde{z}}^{1/\nu }}{L}(1 & +8a\pi \tilde{R}(\left[erfc\left[\frac{a}{\sqrt{2}R_{x}}\right]-\frac{R_{x}}{a}\sqrt{\frac{2}{\pi }}e^{-\frac{a^{2}}{2R_{x}^{2}}}\right]\;\;\\ \; & -\;\;2[erfc\left[\frac{\sqrt{2}a}{R_{x}}\right]-\frac{R_{x}}{a\sqrt{2\pi }}e^{\frac{-2a^{2}}{R_{x}^{2}}}])) \end{array}$$
3. On page 16 symbol ‘cc’ before $<\rho_{\lambda }^{\left(DD\right)}\left(\tilde{z}\right){>}_{st}$ in the Equation (44) should be removed and looks like:
  (44) $$\begin{array}{cc} <\rho_{\lambda }^{\left(DD\right)}\left(\tilde{z}\right){>}_{st}=B\frac{{\tilde{z}}^{1/\nu }}{L}(1 & -2\sqrt{5\pi }\tilde{R}a[\left(\frac{61R_{x}}{7a}+\frac{1584}{175}\frac{a}{R_{x}}\right)e^{-\frac{4a^{2}}{5R_{x}^{2}}}\\ & -\left(\frac{155R_{x}}{7a}+\frac{10,272}{175}\frac{a}{R_{x}}\right)e^{-\frac{16a^{2}}{5R_{x}^{2}}}]) \end{array}$$
4. On page 21 Appendix B symbol ‘ccccc’ before ${\tilde{G}}_{\|}\left(p;z,z^{\prime };\mu_{0},c_{1_{0}},c_{2_{0}},L\right)$ in the Equation (A5) should be removed and looks like:
  (A5) $$\begin{array}{cc} {\tilde{G}}_{\|}\left(p;z,z^{\prime };\mu_{0},c_{1_{0}},c_{2_{0}},L\right) & =\frac{1}{2\kappa_{0}}(\left(\kappa_{0}^{2}+\kappa_{0}\left(c_{1_{0}}+c_{2_{0}}\right)+c_{1_{0}}c_{2_{0}}\right)e^{\kappa_{0}L}\\ \; & -\left(\kappa_{0}^{2}-\kappa_{0}\left(c_{1_{0}}+c_{2_{0}}\right)+c_{1_{0}}c_{2_{0}}\right)e^{-\kappa_{0}L}{)}^{-1}\\ \; & (\left(\kappa_{0}^{2}+\kappa_{0}\left(c_{1_{0}}+c_{2_{0}}\right)+c_{1_{0}}c_{2_{0}}\right)e^{\kappa_{0}(L-|z-z^{\prime }\left|\right)}\\ \; & +\left(\kappa_{0}^{2}-\kappa_{0}\left(c_{1_{0}}+c_{2_{0}}\right)+c_{1_{0}}c_{2_{0}}\right)e^{-\kappa_{0}(L-|z-z^{\prime }\left|\right)}\\ \; & +\left(\kappa_{0}^{2}+\kappa_{0}\left(c_{2_{0}}-c_{1_{0}}\right)-c_{1_{0}}c_{2_{0}}\right)e^{\kappa_{0}\left(L-z-z^{\prime }\right)}\\ \; & +\left(\kappa_{0}^{2}-\kappa_{0}\left(c_{2_{0}}-c_{1_{0}}\right)-c_{1_{0}}c_{2_{0}}\right)e^{-\kappa_{0}\left(L-z-z^{\prime }\right)}) \end{array}$$
5. On page 22 Appendix C symbol ‘cc’ before $<\rho_{\lambda }^{\left(DN\right)}\left(\tilde{z}\right)>$ in the Equation (A8) should be removed and looks like:
  (A8) $$\begin{array}{cc} <\rho_{\lambda }^{\left(DN\right)}\left(\tilde{z}\right)> & =\frac{{\left(2R_{g}\right)}^{1/\nu }}{L_{0}}\frac{\mathrm{IL}\int_{0}^{L}dzG\left(;z,\tilde{z}\right)G\left(;\tilde{z},z\right)}{\mathrm{IL}\int_{0}^{L}dzG\left(;z,z\right)}\\ \; & \approx 2\frac{{\tilde{z}}^{1/\nu }}{L}+\frac{\tilde{z}}{y^{2}}\frac{\left(e^{-4y^{2}}-e^{-16y^{2}}\right)}{\left(1-2e^{-4y^{2}}+2e^{-16y^{2}}\right)} \end{array}$$

The changes do not influence substantive meaning and the obtained scientific results. The original article has been updated.
